# Evaluation of preoperative intra-aortic balloon pump in coronary patients with severe left ventricular dysfunction undergoing OPCAB surgery: early and mid-term outcomes

**DOI:** 10.1186/1749-8090-4-39

**Published:** 2009-07-27

**Authors:** Zhibing Qiu, Xin Chen, Ming Xu, Yingshuo Jiang, Liqiong Xiao, LeLe Liu, Liming Wang

**Affiliations:** 1 Department of Thoracic and Cardiothoracic Surgery, Nanjing First Hospital affiliated to Nanjing Medical University, Nanjing Heart Institute, Changle Rd. 68, 210006 Nanjing, PR China

## Abstract

**Background:**

The purpose of the present study was to evaluate the safety and the cost-effectiveness of using preoperative IABP as support compared with postoperative IABP treatment in coronary patients with severe left ventricular dysfunction (SLVD) who is undergoing off-pump coronary artery bypass surgery (OPCAB), including early outcomes, hospital mortality and morbidity, and mid-term follow-up outcomes.

**Methods:**

Between March 2000 and December 2008, we prospectively and randomly studied the insertion of preoperative IABP in 115 (7.4%) and postoperative IABP in 106 (6.8%) of the 1560 consecutive patients. Group A is preoperative IABP therapy. Group B is postoperative IABP therapy.

**Results:**

There was no significant difference in the number of grafts used between the two groups. Completeness of revascularization did not differ between the two groups. The statistically significant difference was hospital mortality (2.6% in group A vs. 3.8% in group B) (*p *< 0.05). And there was significant reduction in postoperative low cardiac output, malignant arrhythmia, acute renal failure and length of stay in ICU in group A, compared with group B (*p *< 0.05). In the two groups, six-, 12-, 24- and 48-month survival rates were similar. In the study the degree of improvement in angina and quality of life did not differ significantly between the two groups.

**Conclusion:**

The use of preoperative IABP in SLVD patients undergoing OPCAB is of safety and effectiveness. The combined use of preoperative IABP and OPCAB allows complete revascularization in SLVD patients with an important reduction in operative mortality and excellent mid-term results.

## Background

Despite improvements in medical therapies and surgical techniques, the management of coronary patients with severe left ventricular dysfunction (SLVD) (ejection fraction [EF] = 0.35) is still challenging[1,2]. For this population, CABG is associated with higher postoperative morbidity and mortality compared with patients with normal left ventricular function[3]. Off pump coronary artery bypass surgery (OPCAB) has theoretical and practical advantages over conventional coronary artery bypass grafting (CCABG)[4,5].

Also in high risk coronary patients, OPCAB is an attractive alternative, but due to hemodynamic instability this cohort of patients has usually been operated using some circulatory support. The elective use of intra-aortic balloon pump (IABP) in these patients may prevent this and thus avoid the institution of CPB with its attendant risks which include inflammation and global ischemia[6,7] The use of pre- and postoperative IABP has been suggested, but not yet fully explored[8,9]. The purpose of the present study is to evaluate, in a prospective and randomized manner, the safety and the effectiveness of using preoperative IABP as support compared with postoperative IABP treatment.

## Patients and methods

### Patients and inclusion criteria

The study was approved by the Institutional Review Board in March 3, 2000, and informed consent was obtained from all patients. Since March 2000, OPCAB has been the technique of choice at our center for all isolated coronary procedures, and it was used in 1560 consecutive patients. Between March 2000 and December 2008, we prospectively studied the insertion of preoperative Datascope system IABP in 115 (7.4%) consecutive patients with poor preoperative left ventricular ejection fraction (LVEF) = 35%. During the same period, the IABP was inserted during or after the operation in 106 (6.8%) of the 1560 patients. The LVEF of all patients was calculated from echocardiography assessment performed by an independent cardiologist.

#### Inclusion criteria

Adult patients with coronary artery disease (CAD) admitted for elective or urgent myocardial revascularization by OPCAB and classified as patients with severe left ventricular dysfunction according to definition given above and judged suitable for OPCAB surgery by the responsible surgeon were included in study. Patients with moderate and severe mitral regurgitation MR were excluded because of our practice to perform mitral valve repair/replacement in these patients at the initial procedure.

The patients fulfilling the above criteria were randomized into either of two groups by lottery (prepared closed envelopes containing the group assignment): Group A–preoperative IABP therapy, started 24 hour(h) prior to induction of anesthesia, followed by continuous IABP during the entire procedure as well as postoperatively. Group B–postoperative IABP therapy, inserted during or after the operation. Postoperative IABP treatment was initiated if fulfilling definitions stated: when CI cannot be maintained at a level greater than 2.0 L/min/m2, despite pharmacological support with epinephrine equal or more than 0.5 *μ*g/kg/min, dobutamine equal or more than 10 *μ*g/kg/min and amrinonum equal or more than 0.5 mg/kg bolus dose, an IABP treatment was indicated.

All preoperative clinical, operative data and follow-up data were entered into a computer data base. Definitions were laid prior to the start of the study and had not been changed during the study period.

All the patients had multi-vessel coronary artery disease with severe left ventricular dysfunction and other patients' characteristics for each group are presented in Table 1. No significant differences were found between two groups in baseline variables such as age or comorbidities. The overall predicted risk according to the EuroScore was similar between the two groups.

**Table 1:** Preoperative Characteristics

Variables	Group A (n = 115) N (%)	Group B (n = 106) N (%)	P value
Female sex	42(36.5)	32 (30.2)	0.42
Age > 65 years	78 (67.8)	80 (75.5)	0.11
Symptom status(stable)	66 (57.4)	70 (66.0)	0.65
Diabetes	35 (30.4)	36 (34.0)	0.77
Hypertension	70 (60.9)	79 (74.5)	0.12
Hypercholesterolemia	85 (73.9)	76 (71.7)	0.88
Smoking	86 (74.8)	72(67.9)	0.61
Chronic Renal dysfunction	4 (3.5)	3 (2.8)	0.08
Chronic Gastritis	9 (7.8)	10 (9.4)	0.11
COPD	16 (13.9)	18 (17.0)	0.47
Peripheral vascular disease	7(6.1)	5 (4.7)	0.21
MI	73 (63.5)	79 (74.5)	0.06
Angina (CCS III/IV)	95 (82.6)	84 (79.2)	0.21
Dyspnea (NYHA III/IV)	55(47.8)	42 (39.6)	0.18
Redo CABG	2 (1.7)	1(0.9)	0.07
Prior CVA	5 (4.3)	3 (2.8)	0.11
BMI > 30 kg/m^2^	45 (39.1)	35 (33.0)	0.45
Aspirin<10 days	96 (83.5)	78(73.6)	0.17
Previous failed PTCA	8 (7.0)	8 (7.5)	0.64
Parsonnet score >10	73 (63.5)	73 (68.9)	0.37
Left main disease	20 (17.2)	21 (19.8)	0.58
Mean Diseased vessels	3.20 ± 0.36	3.42 ± 0.52	0.85
Mean EuroSCORE	12.58 ± 3.05	13.37 ± 3.13	0.26
Mean LVEF(%)	29.62 ± 5.32	30.04 ± 4.27	0.36

CCS = Canadian Cardiovascular Society score; COPD = chronic obstructive pulmonary disease; MI = myocardial infarction; CVA = cerebrovascular accident; GI = gastrointestinal; BMI = body mass index; PTCA = percutaneous transluminal coronary angioplasty;

### Intra-aortic balloon pump (IABP)

The IABP catheter used was 8 F 34 ml balloon Percor STAT-DL Catheter (Datascope Corp, Fairfield, NJ) connected to a Datascope portable computerized console (Datascope), placed using percutaneous insertion technique via femoral artery. In group A, preoperative insertion was normally performed in the anesthesia preparation room in the operating room (OR) prior to induction of anesthesia. Group B patients received their IABP in the operating room if fulfilling definitions stated above. There was no failure of percutaneous placement of the IABP, while using the guide-wire. Unless heparin was contraindicated, patients were therapeutically anticoagulated with heparin after IABP placement. Patients returning from the operating room with an IABP in place were given Dextran until the mediastinal drainage removed (usually within 24 h) for anticoagulation.

IABP therapy for group A was continued postoperatively only when indicated based on restoration of hemodynamic stability maintaining a CI greater than 2 L/min/m2, with only minimal pharmacological inotropic support. The same definition was used for termination of postoperative IABP for Group B.

### Surgical procedure

Standard intraoperative monitoring techniques were used. A CPB circuit was on stand-by for all cases. All procedures were performed through a median sternotomy. After the conduits (internal thoracic arteries, the radial artery, and saphenous vein) were harvested, heparin was administered to maintain an activated clotting time greater than 250 seconds. Three deep left pericardial sutures were used for cardiac exposure, and a suction device (Octopus Evolution, Medtronic, Minneapolis, MN) was used for stabilization of the coronary arteries. A shunt (Chase Medical, Richardson, TX) was inserted in the coronary artery during all anastomoses to avoid ischemic damage and perioperative rhythm disturbances. A blower/mister was systematically used to obtain a bloodless operative field and perfect visualization of the coronary artery. The left anterior descending artery was revascularized first in all patients. In cases of vein grafting, the proximal anastomosis of the vein on the aorta was performed before the distal anastomosis under side clamping. The IABP was placed on stand-by during the proximal anastomosis. A cell-saving device was used in all patients.

Preoperative, operative, and postoperative variables between the groups were investigated, including hospital mortality and morbidity rates, such as neurologic events, perioperative infarction, renal insufficiency, rhythm disturbances, respiratory failure, hospital length of stay, in-hospital death and reoperations for excessive postoperative bleeding or ischemia.

### Mid-term follow up

Mid-term follow-up was achieved by direct telephone contact with the patient, family, primary care physician, or cardiologist. If necessary, additional information was obtained from patient's hospital and office records. The endpoints were return to work rates, disease specific quality of life (QOL) using Seattle Angina Questionnaire (SAQ). The SAQ is a 19-item disease-specific self-administered questionnaire assesses physical limitation, angina frequency, treatment satisfaction and disease perception/QOL[10]. Higher scores on SAQ subscales indicate better levels of functioning. Patients were classified as employed at baseline if they were working full- or part-time or were on sick leave, with expectation of returning to work for such patients was defined as working full- or part-time at follow-up.

### Statistics

Results are expressed as the mean value ± standard deviation. Microsoft Excel was used for all statistical data processing. Data were examined univariately by the ANOVA test for continuous variables, and the χ^2 ^analysis was used for discrete data. After completion of the propensity model, a propensity score for mortality was calculated from the logistic equation for each patient. Then, on this basis, patients were sorted by propensity and compared within five quintiles [11]. Survival curves were drawn on an actuarial basis using the Kaplan-Meier technique. Statistical significance was considered at a value of *p *< 0.05. Data were analyzed using SPSS 13.0 (SPSS).

## Results

### Perioperative characteristics

There was no significant difference in the number of grafts used between the two groups (Table 2). Group A patients received 3.3 ± 0.6 grafts per patient, while group B patients had 3.1 ± 0.5 (p = 0.36). There was no significant difference in the use of left internal mammary artery (LIMA), right internal mammary artery and radial arterial grafts in the two groups. The distribution of distal anastomosis was similar between the two groups: 3.8 ± 0.5 in group A, and 3.6 ± 0.3 in group B (*p *= 0.24). Completeness of revascularization did not differ between the two groups (group A 87.3% vs. group B 86.9%; p = 0.18). Coronary thrombendarterectomy was required in 16(7.2%) cases, without group differences.

**Table 2:** Operative Data

Variables	Group A (n = 115) N (%)	Group B (n = 106) N (%)	p Value
Number of grafts	Mean = 3.3 ± 0.6	Mean = 3.1 ± 0.5	0.36
Grafts, n(%)			
2 SVG	18 (15.6)	19(17.9)	0.14
3 SVG	71 (61.7)	67(63.2)	0.26
4 SVG	26 (22.6)	20 (18.9)	0.13
LIMA	95 (82.6)	85(80.2)	0.64
RIMA	2 (1.7)	3 (2.8)	0.08
Radial	25 (21.7)	20(18.8)	0.19
Distal anastomosis(n)	Mean = 3.8 ± 0.5	Mean = 3.6 ± 0.3	0.24
Complete revascularization	100 (87.0)	86 (81.1)	0.18

SVG = saphenous vein graft; LIMA = left internal mammary artery; RIMA = right internal mammary artery;

### Hospital Mortality

The only statistically significant difference was hospital mortality (2.6% in group A vs. 3.8% in group B), which showed significantly lower in group A (*p *= 0.031). In the group A, the cause of death was right ventricular failure in one patient with preoperative massive right ventricular infarction, and multiple organ failure in two patient. But in the group B, the cause of death was cardiac arrest in one patient, and multiple organ failure in three patients.

### Hospital Morbidity

There was no significant difference in neurologic events, postoperative infarction, wound infection, respiratory failure, new-onset atrial fibrillation and reoperations for excessive bleeding between two groups (Table 3). However, there was significant reduction in postoperative low cardiac output, malignant arrhythmia, acute renal failure and length of stay in ICU in group A, compared with group B. Only 12 patients(10.4%) in group A had postoperative low cardiac output in contrast 20 patient(18.9%) in group B (*p *< 0.05). The ventricular arrhythmia occurred in seven patient (6.1%) in group A and 13 patients(12.3%) in group B (*p *< 0.05). The acute renal failure occurred in eight patients(6.9%) in group A and 12 patients(11.3%) in group B (*p *< 0.05). The prolonged stay(≥3 days) in the ICU was 33.9% in group A and 54.7% in group B (*p *< 0.05). The mean postoperative IABP time was 2.2 ± 0.7 days in group A and 3.6 ± 1.3 days in group B (*p *< 0.05).

**Table 3:** Hospital morbidity

Variables	Group A (n = 115) N (%)	Group B (n = 106) N (%)	p value
Low Cardiac output	12 (10.4)	20(18.9)	< 0.05
Reoperation for bleeding	8 (6.9)	6 (5.7)	0.08
IABP complication	0	0	-
Postoperative MI	2 (1.7)	2 (1.9)	0.54
New-onset atrial fibrillation	50 (43.5)	53 (50.0)	0.19
Ventricular arrhythmia	7 (6.1)	13 (12.3)	< 0.05
Pleural effusion	78 (67.8)	76(72.0)	0.21
Wound infection	3 (2.6)	2 (1.9)	0.18
Acute Renal failure	8 (6.9)	12(11.3)	< 0.05
Prolonged ventilatory support ≥ 24 h	38 (33.0)	42 (39.6)	0.16
Stroke and cerebrovascular accident	2 (1.7)	3 (2.8)	0.07
Prolonged stay (≥3 days) in ICU	39(33.9)	38(54.7)	< 0.05
Postoperative IABP time(Days)	2.2 ± 0.7	3.6 ± 1.3	< 0.05

### IABP-Related Morbidity

Vascular complications occurred in 2 patients of group A and in 3 patients of group B. There were two retroperitoneal heamatomas and one distal limb ischemia (which resolved with balloon removal). There was no IABP-related mortality in group A or group B. No infections occurred in either group. There was no complication related to the use of heparin perioperatively in these patients.

### Mid-Term Follow-Up

**Figure 1:**
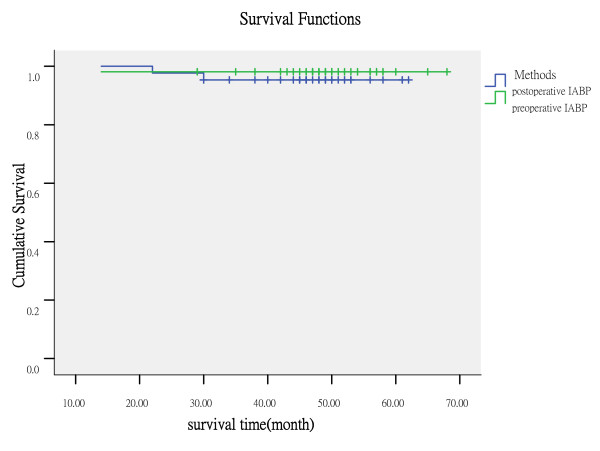
The Kaplan Meier survival curve.

Follow-up was achieved for survivors (mean follow-up time: 48.4 ± 11.6 months). Two patients were lost to follow-up in the group A, and three patients were lost in the group B. Three patients died during follow-up: one patient in group A from pulmonary neoplasm after 15 months, and one patient in group B from terminal renal insufficiency after 13 months, another one patient in group B from cerebrovascular accident after 3 years. No major cardiac event due to incomplete revascularization was reported during follow-up. There was no hemorrhaging, thromboembolic complications, or stenosis during follow-up. The Kaplan Meier survival curve, including perioperative deaths, was presented in Fig. 1. In the two groups, six-, 12-, 24- and 48-month survival rates were similar.

All 81 survivors completed the SAQ questionnaire (response rate 100%). At follow-up, physical limitation, angina stability, angina frequency, treatment satisfaction and quality of life were comparable between the two treatment groups. In comparison with an age- and sex-matched standard population, Group A and Group B patients were impaired (score<85) in physical limitation and in quality of life (Table 4).

**Table 4:** Quality of life

Variables*	Group A (n = 110)	Group B (n = 100)	p Value
Physical limitation	77 ± 11	79 ± 12	0.88
Angina stability	90 ± 13	92 ± 15	0.86
Angina frequency	85 ± 14	88 ± 16	0.52
Treatment satisfaction	69 ± 9	71 ± 10	0.35
Quality of life	74 ± 10	78 ± 13	0.24
Return to work	31/110(28.2%)	25/100(22.7%)	0.31

*Quality of life assessment by Seattle Angina Questionnaire disease – specific instrument: score range from 0(worst) to 100(best health status)

## Discussion

With the enormous growth of interventional cardiology in recent years, patients coming to surgery for coronary artery bypass grafting are often at the end-stage of their disease, with severely impaired LV function. Management of patients with SLVD caused by coronary artery disease remains a challenge. The increased popularity and success of OPCAB grafting during the past decade seems to be a good surgical option. Good surgical results were obtained from different study [12,13].

Earlier reports have shown that use of preoperative IABP therapy can reduce myocardial ischemia and therefore improve outcome in high-risk patients undergoing CABG with the use of CPB[14]. Recent reports have indicated that pre- and perioperative IABP therapy facilitates manipulation of the heart with maintained hemodynamic stability and with reduced myocardial oxygen demand in high-risk patients undergoing OPCAB surgery [15]. The purpose of this study is to evaluate whether the use of preoperative intra-aortic balloon pump treatment can improve the outcome after surgical myocardial revascularization, and to evaluate whether this additional treatment is safety and effectiveness.

Indications for preoperative insertion of IABP has usually been reserved for patients with angina refractory to maximal medical therapy or for those with very low cardiac output preoperatively[5]. Our selection criteria was specifically designed to aggressively use the IABP preoperatively in an effort to avoid the complications and mortality associated with intraoperative or postoperatively insertion(delayed insertion). We designed our study to prospectively compare the results in two groups of patients using a prospective and randomized manner.

The number of anastomoses per patient in the study was similar to that in our current practice, as complete revascularization remains the principal advantage of cardiac operations and is essential in these patients, which corresponds with results of earlier reports[5,16]. Our current practice is to perform systematic off-pump operations for all isolated coronary procedures, representing a group of 1560 consecutive patients for eight years.

This study compares mortality between the two groups of patients receiving IABP. It is probably not surprising that hospital mortality is lower for group A cases than for group B cases. The improved hospital mortality in the preoperative IABP group compared with postoperative IABP group most likely reflects the avoidance of progressive cardiac dysfunction before insertion. Moreover, in the study the benefit of OPCAB with IABP extended beyond the early risk phase (the first year) and was not associated with an increased mortality in the mid-term interval. Due to the high risk of preoperative severely impaired LV function, the mortality is rather high for the series of patients. And most of the deaths occurred in the early stage of the study, related to our early experience. Three patients died during follow-up, due to the non cardiac events.

In the present prospective randomized study, we have clearly demonstrated that pre- and postoperative IABP therapy are equally effective and safe, because of significant decreases of the risk for hemodynamic instability and conversion to CPB in coronary patients with SLVD. There were no statistically significant differences in most clinical outcome parameters between group A and group B, thus indicating that the IABP therapy is effective as well, as earlier documents[17]. However, it is notable that the prolonged stay in ICU as well as IABP support time was significantly shorter for group A than group B. In the present series, there were less IABP-related complications that could be explained by the usage of small sized balloon catheters (8F), an experienced team, short duration of therapy and close surveillance.

Patients who received a preoperative IABP (group A) have a lower rate of postoperative low cardiac output and ventricular arrhythmia than those receiving postoperative IABP (group B). This difference probably highlighted the benefits of preoperative IABP in term of improved myocardial oxygen supply/demand ratio, redistribution of blood flow toward areas of ischemic myocardium, hemodynamic stability during induction and prebypass and improved graft flow postbypass[18]. In the study, eight patients (6.9%) in group A and 12 in group A (11.3%) developed acute renal dysfunction. This demonstrates that preoperative elective IABP counterpulsation in high-risk OPCAB surgery leads to a significant reduction in the incidence of acute renal dysfunction and the need for hemofiltration, which supports the finding of Vohra, et al [19]. It has been proposed that the use of preoperative IABP may lead to minimization of low-flow episodes with avoidance of subsequent end organ dysfunction, especially OPCAB patients with SLVD.

Once the postoperative phase is achieved, our mid-term follow-up shows excellent clinical results, with no subsequent revascularizations due to incomplete procedures, thus confirming the adequacy of the revascularization. On follow-up, most of the patients were free from angina, rehospitalization and recatheterization both the two groups postoperatively. In our study the degree of improvement in angina and quality of life did not differ significantly between the two groups. The SAQ questionnaire is more sensitive and responsive to detect changes in angina-related health status than other generic measures and it provides more clinically relevant information with respect to the disease of interest [10]. Though the survival curve of the preoperative IABP therapy group patients appears to indicate slightly better early survival than the postoperative IABP therapy group cohort, no statistical difference was demonstrated.

## Conclusion

The present study is limited by the small size of its population. Further studies with a larger population are needed to single out a group of heart failure patients, which could benefit most from using intra-aortic balloon pump as a supportive or bridge therapy. In conclusion this study has demonstrated a beneficial effect of preoperative IABP treatment in coronary artery patients with SLVD undergoing OPCAB.

## Competing interests

The authors declare that they have no competing interests.

## Authors' contributions

QZB and CX had helped with design of the study, data interpretation and in writing of the paper. XM has made the statistical analysis and took part in the writing process. QZB also took part in the correction of the manuscript according to the reviewers suggestions. JYS and WLM had helped in gathering patient information and performed graphic measurements. XLQ and LLL performed graphics and tables and added comments to the paper. All authors read and approved the final manuscript.
